# A preliminary study of radioulnar wrist compression in improving patient-reported outcomes of carpal tunnel syndrome

**DOI:** 10.1186/s12891-022-05943-0

**Published:** 2022-11-09

**Authors:** Zong-Ming Li, Emily L. Grandy, Lenicia Jenkins, Carli Norman, James Bena, Juliet Hou, Peter J. Evans, William H. Seitz, C. Kent Kwoh

**Affiliations:** 1 Departments of Biomedical Engineering, Cleveland Clinic, Cleveland, OH USA; 2 Physical Medicine and Rehabilitation, Cleveland Clinic, Cleveland, OH USA; 3 Department of Orthopaedic Surgery, Cleveland Clinic, Cleveland, OH USA; 4 Hand Research Laboratory, Departments of Orthopaedic Surgery and Biomedical Engineering, University of Arizona, Tucson, AZ USA; 5 Department of Quantitative Health Sciences, Cleveland Clinic, Cleveland, OH USA; 6 Arthritis Center, University of Arizona, Tucson, AZ USA

**Keywords:** Carpal tunnel syndrome, Wrist compression, Noninvasive treatment, Clinical symptom

## Abstract

Previous studies have shown radioulnar wrist compression augments carpal arch space. This study investigated the effects of radioulnar wrist compression on patient-reported outcomes associated with carpal tunnel syndrome. Subjects underwent thrice-daily (15 min each time 45 min daily) wrist compression over 4 weeks with an additional four weeks of follow-up without treatment. Primary outcomes included Boston Carpal Tunnel Questionnaire symptom and functional severity scales (SSS and FSS) and symptoms of numbness/tingling based on Visual Analog Scales. Our results showed that radioulnar wrist compression improved SSS by 0.55 points after 2 weeks (*p* < 0.001) and 0.51 points at 4 weeks (*p* < 0.006) compared to the baseline scale. At the four-week follow-up, SSS remined improved at 0.47 points (*p* < 0.05). Symptoms of numbness/tingling improved at two and 4 weeks, as well as the follow-up (*p* < 0.05). Hand motor impairment such as weakness had a lower frequency across carpal tunnel syndrome sufferers and does not significantly improve (*p* > 0.05). Radioulnar wrist compression might be an effective alternative treatment in improving sensory related symptoms in patients with mild to moderate carpal tunnel syndrome.

## Introduction

Despite the widespread burden of carpal tunnel syndrome, clinical management of CTS can be challenging for both physicians and patients. Current treatment options fall into either conservative or surgical intervention categories. Non-invasive methods to alleviate CTS symptoms include nonsteroidal anti-inflammatory drugs, corticosteroids, stretching, splinting, massage, and laser treatment [1]. Carpal tunnel release surgery to transect the transverse carpal ligament is currently considered the gold standard solution to decompress the median nerve. Unfortunately, the surgical procedure unavoidably disrupts essential anatomical, biomechanical and physiological functions of the wrist [2] and reduced grip strength, pillar pain, carpal bone instability, scar tissue formation, and perineural fibrosis [3].

In an attempt to decompress the median nerve non-surgically, we developed a biomechanical approach to augment the carpal arch. When an external force is applied and directed across the width of the wrist in the radioulnar direction, the distance between the trapezium and hook of hamate narrows, bowing the transverse carpal ligament palmarly. The mechanisms of wrist compression for carpal arch space augmentation and its implications to decompress the median nerve have been demonstrated through geometric modeling [4], finite element analysis [5], and in vitro experiments [6], and in vivo study [7]. In particular, radioulnar wrist compression has also been shown to alleviate median nerve compression and restore impaired neurophysiological and biomechanical functions of the nerve in CTS patients, as indicated by improvements in median nerve flattening [7], nerve mobility [8], cross sectional area [9], and distal motor latency [10]. However, the clinical efficacy of patient-reported outcomes is unknown.

The purpose of this study was to investigate the effects of radioulnar wrist compression on patient reported clinical outcomes associated with CTS, including hand symptoms and motor function. Subjects underwent thrice-daily radioulnar wrist compression over a period of 4 weeks, with an additional 4 weeks of follow-up. We hypothesized that radioulnar wrist compression would improve both symptoms and functional deficits associated with CTS over the 4-week intervention period. We further hypothesized that the improvement would be sustained after cessation of the intervention.

## Methods and materials

### Patients and sample size

Twenty-one patients clinically diagnosed with CTS by nerve conduction studies were recruited through physician referrals or patient database. Exclusion criteria included history of hand surgery, carpal tunnel release surgery, systemic disease or condition commonly associated with increased incidence of CTS (e.g. diabetes, thyroid disease, rheumatoid arthritis, fibromyalgia, pregnancy), BMI > 30, and non-English speaking. Enrolled subjects were examined to confirm medical and symptomatic history and elicited positive provocative signs of Tinel’s or Phalen’s maneuver. Patients completed the Edinburgh Handedness Inventory [11] as an objective measure of hand dominance. For patients presenting with bilateral CTS, the more affected hand was treated. In the case of equally severe bilateral CTS, the dominant hand was treated. Each patient completed written informed consent approved by Institutional Review Board of Cleveland Clinic prior to his/her participation.

### Intervention

**Fig. 1:**
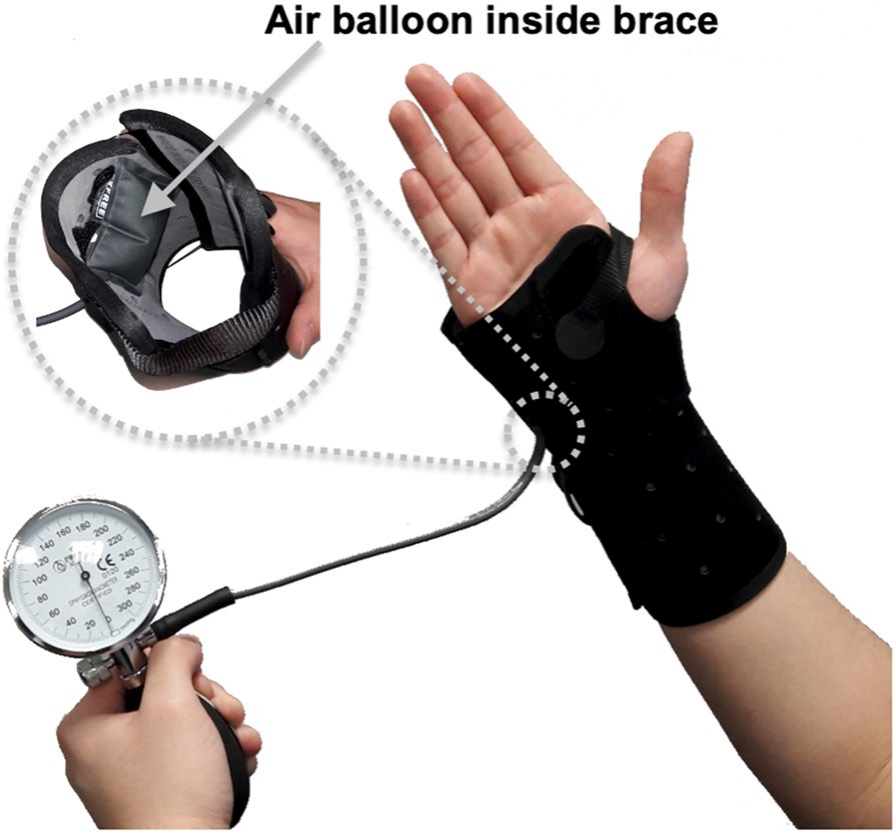
Wearable brace with inflatable balloon bladder in the brace for radioulnar wrist compression

To facilitate self-use, a portable, wearable wrist device was developed to reproduce the biomechanical specifications of our laboratory-based system [7, 12]. The compression device consisted of a thermoformable Exos Wrist Brace with Boa (DJO, Vista, CA), capable of molding to the patient’s wrist for a custom fit in an anatomically neutral position. An air bladder (50 mm × 50 mm) was attached to the inside of the brace. The bladder was centered around the hamate level on the ulnar side of the wrist. To inflate the bladder and apply wrist compression in the radioulnar direction, an aneroid sphygmomanometer dial, bulb, and air valve was attached to the bladder via a rubber hose (Fig. 1).

Patients were trained to perform the wrist compression intervention protocol and instructions for use were provided for at-home reference. During the four-week intervention period, patients performed three radioulnar wrist compression sessions daily. Each session included three 5-minute wrist compression with a 1-minute rest between intervals. A 10 N compressive force was achieved by inflating the bladder to a pre-calibrated pressure of 140 mmHg according to our previous study [7]. After the 4-week intervention, each patient was required to refrain from any other treatment for an additional 4 weeks of follow-up.

### Primary and secondary outcomes

Primary outcome measures included (i) Boston Carpal Tunnel Syndrome Questionnaire (BCTQ) Symptom Severity Scale (SSS), Functional Status Scale (FSS) and Overall Scale [13]; and (ii) Visual Analog Scale (VAS, 0–10) for worst pain, numbness and tingling. Secondary outcome measures included (iii) Semmes-Weinstein monofilament (SWM), (iv) two-point discrimination (2PD), and (v) grip and pinch strength. BCTQ and VAS questionnaires were self-administered. All primary and secondary outcomes were measured at baseline (Week 0), twice during the intervention period (Weeks 2 and 4), and at follow-up (Week 8).

### Statistical comparison

Continuous measures are shown as means with standard deviations. Normality of outcomes was evaluated using Shapiro-Wilk tests. Linear mixed effect models were fit for each outcome. Time was included as a predictor and modeled categorically to allow for non-linear changes over time. An autoregressive correlation structure for repeated measures with heterogeneous variance structure was assumed. Mean levels at each time point with 95% confidence intervals are presented. Analysis was performed using SAS software (Cary, NC). A significance level of 0.05 was assumed for all tests.

## Results

Among the 21 CTS patients were recruited, 5 either dropped out or were withdrawn. Sixteen patients completed the study and their data were used for analyses (Table 1). Of the 16 subjects who completed the study, 11 were female (68.7%). Mean age of all patients was 52.6 years (± 14.0); 14 were right-handed, 1 was left-handed, and 1 was ambidextrous. Based on clinical diagnostics, 6 patients (37.5%) had bilateral CTS.

**Table 1:** Patient information (Mean ± SD)

	Female (*N* = 11)	Male (*N* = 5)
Age	52.7 ± 11.7	52.2 ± 19.8
BMI	26.3 ± 3.1	26.9 ± 2.5
CTS Hand – Right	*N* = 7	*N* = 1
CTS Hand – Left	*N* = 1	*N* = 1
CTS Hand - Bilateral	*N* = 3	*N* = 3

### Primary outcome

**Fig. 2:**
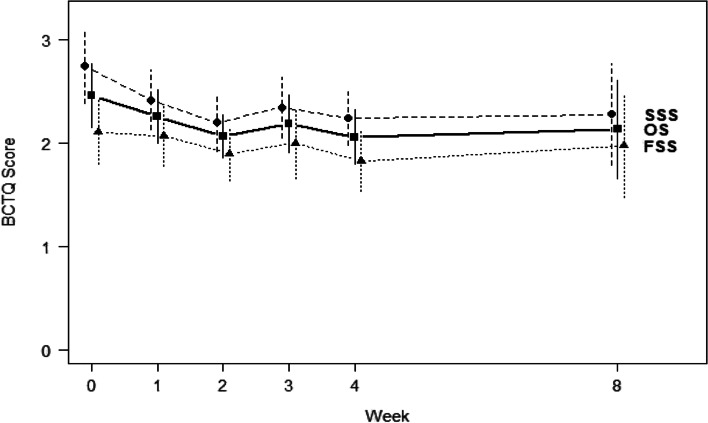
Changes in BCTQ Symptom Severity Scale (SSS), Function Severity Scale (FSS), and Overall Scale (OS) over time with 95% confidence intervals

The overall BCTQ score at baseline were 2.46 ± 0.69 with the severity in the range of mild to moderate. At baseline, SSS averaged 0.64 points higher (i.e. more severe) than FSS. SSS significantly improved by 0.55 points (*p* < 0.001) and 0.51 points (*p* = 0.006) at Weeks 2 and 4 compared to baseline, and a 0.47 points improvement by Week 8 (*p* < 0.05) compared to baseline. On average, FSS improved by 0.21, 0.28, and 0.13 points at Weeks 2, 4, and 8 compared to baseline, respectively, but these improvements were not statistically significant (*p* > 0.103). See Fig. 2 and Table 2.

**Table 2:** Primary outcome scores at Weeks 0, 2, 4, and 8 (Mean ± SD)

	Week 0	Week 2	Week 4	Week 8 Follow up
BCTQ SSS	2.75 ± 0.67	2.20 ± 0.56	2.24 ± 0.50	2.28 ± 0.87
BCTS FSS	2.11 ± 0.67	1.90 ± 0.51	1.83 ± 0.49	1.98 ± 0.81
VAS Pain	5.31 ± 3.66	3.94 ± 2.72	4.06 ± 2.95	3.69 ± 3.48
VAS Numbness	6.25 ± 2.70	4.06 ± 2.05	3.94 ± 2.17	3.69 ± 2.94
VAS Tingling	5.44 ± 3.39	3.25 ± 2.21	3.38 ± 2.66	2.81 ± 2.56

Pain, numbness, and tingling had all significantly improved by Week 2 of intervention (*p* < 0.05). By Week 4, numbness and tingling remained improved relative to baseline (*p* < 0.05), but improvements in pain at this time point did not reach significance (*p* = 0.120). At Week 8 follow up, numbness and tingling had improved relative to baseline (*p* < 0.05), but pain scores had not significantly changed (*p* = 0.089).

### Secondary outcome

For the secondary outcome measures, SWM score improved significantly by Week 2 (*p* = 0.049), Week 4 (*p* = 0.031), and follow up Week 8 (*p* = 0.033) compared to baseline. There were no significant differences across time points for 2PD or pinch/grip strength (*p* > 0.05). See Table 3.

**Table 3:** Strength and sensory outcomes (Mean +/− SD)

	Week 0	Week 2	Week 4	Week 8
SWM	3.35 ± 0.54	3.16 ± 0.48	3.14 ± 0.44	3.06 ± 0.44
2PD	3.63 ± 1.23	3.64 ± 1.29	3.67 ± 1.34	3.31 ± 1.21
Pinch Strength, N	52.8 ± 18.6	55.3 ± 15.8	53.2 ± 16.1	55.3 ± 15.4
Grip Strength, N	246.7 ± 120.1	258.9 ± 98.1	257.2 ± 94.6	255.7 ± 84.0

*2PD* Two Point Discrimination, *SWM* Semmes-Weinstein Monofilament

## Discussion

Over a four-week period, during which patients underwent at-home radioulnar wrist compression three times daily, symptom severity noticeably improved. Sensory symptoms, as well as specific measures for pain, numbness, and tingling, markedly improved after just 2 weeks. Additional improvements were detected in SSS, numbness and tingling after a further 2 weeks of daily intervention. Tactile sensation as measured by Semmes-Weinstein monofilament also improved by the second week of intervention. Functional symptoms, including strength (pinch and grip), and overall hand function did not noticeably recover during or after intervention. Four weeks after cessation, Boston Questionnaire SSS as well as numbness and tingling remained improved compared to baseline.

Overnight splinting is often recommended for CTS patients who suffer from nighttime waking. Studies evaluating the effectiveness of regular overnight splinting showed decreases in Boston Questionnaire Overall Scale over a period of 4 weeks (− 0.29) [14], and 6 weeks (− 0.36) [15]. In comparison, radioulnar wrist compression intervention for CTS decreased overall score the most (− 0.40), and did so in the shortest time period of 2 weeks. During 2 weeks of radioulnar wrist compression, improvements to SSS (− 0.51) surpassed those of nighttime splinting for 4 weeks (− 0.38) [14], and 6 weeks (− 0.48) [15]. Notably, nighttime splinting requires approximately 8 hours of nightly wear, while radioulnar wrist compression required only 45 minutes of total wear time (15 minutes, thrice daily), suggesting that our intervention may be the more powerful strategy. Functional status scale was not significantly affected in either nighttime splinting study, which corroborated our results.

Previous studies showed that corticosteroid injection was superior in clinical effectiveness as measured by Boston Questionnaire overall scale at 4 weeks (− 0.44) [14] and 6 weeks (− 0.67) [15]. Completely noninvasive radioulnar wrist compression lessened overall comparably within 2 weeks (− 0.40) and was maintained even after 4 weeks without any intervention (− 0.32 compared to baseline). These results may be explained by the biomechanical manipulation of the carpal tunnel during radioulnar wrist compression intervention. While both night splinting and radioulnar wrist compression use wrist bracing systems, only radioulnar wrist compression augments space within the tunnel, lessening constraints on the median nerve. Although the area underneath the carpal arch is elevated only temporarily (thrice daily) during the course of this intervention, our results suggest that even incremental pressure alleviation may have a noticeable effect on symptom resolution.

Once median nerve compromise has progressed to clinically severe symptoms, or when noninvasive interventions fail, surgical solutions are recommended. The goal of carpal tunnel release is to prevent further progression of the disease by expanding carpal tunnel volume by transecting the transverse carpal ligament (TCL). Similarly, radioulnar wrist compression aims to raise the carpal arch, increasing the cross-sectional area of the carpal tunnel, thereby lessening symptoms of CTS. Previous literature identified the clinically significant BCTQ Scale change for carpal tunnel release surgery to be 0.47 [16]. Interestingly, radioulnar wrist compression intervention for CTS all but achieved this clinically meaningful change after only 2 weeks of 45-minute per day application (− 0.40 overall), and surpassed this target by the end of the intervention (− 0.51, Symptom Severity Scale). Only moderate-quality evidence indicates surgical intervention is superior to splint or steroid injection at 6 months [17]; although the surgical group showed better nerve conduction outcomes at 6 months post-surgery, no significant differences were observed at 3 or 12 months. Other studies have reported higher surgical failure in elderly patients with longstanding disease, neurological deficits, and negative Phalen’s test [18, 19]. For these underserved patients, who often present with more severe CTS symptoms for similar length of history, and for whom surgery might not be a viable option, noninvasive radioulnar wrist compression intervention may offer much needed symptom relief.

As we’ve demonstrated, through geometric modeling [4], in vitro studies [6], and finite element analysis [5], carpal arch width narrowing via radioulnar wrist compression is associated with palmar bowing of the transverse carpal ligament, leading to increased arch height and area. This method of carpal tunnel area expansion maintains the tunnel’s structural integrity while also creating more space for the median nerve. Previous in vivo studies confirmed that the carpal arch can be non-invasively augmented by applying compressive force across the wrist, and that doing so improves median nerve morphology in patients with CTS [7]. A 10 N force applied to the wrist in the radioulnar direction was also shown to restore impaired neurophysiological and biomechanical functions of the median nerve in CTS sufferers; after 2 weeks of thrice-daily compression, nerve mobility [8], cross sectional area [9], and distal motor latency [10] each improved. The present study aimed to demonstrate the effects of radioulnar wrist compression on patient-reported symptomatology and functional performance of the hand over a period of 4-week intervention and 4-week follow-up.

In this study, patients likely experienced the greatest relief from sensory symptoms, including pain, numbness, tingling and dermal sensation, over functional symptoms due to the fact that sensory nerve fibers are predominantly affected in CTS [20]. The most characteristic manifestation of CTS is the sensation of numbness and tingling (paresthesia) [21]. During 4 weeks of daily radioulnar wrist compression, patients in this study noted that symptoms of paresthesia were more greatly influenced compared to symptoms of pain. Although pain also occurs frequently in CTS sufferers, it is less specific in its presentation [21]. Pain evaluation in this study showed that by the second week of the intervention symptoms had markedly improved, and remained stable throughout the remaining 2 weeks of intervention such that no significant differences in pain were observed between Weeks 2 and 4.

While sensory symptoms are the main complaint in CTS, about half of CTS patients also experience motor deterioration, resulting in hand weakness and difficulty grasping small objects [22]. In this study, functional motor symptoms, and specifically pinch and grip strength, were reported as being less severe than sensory symptoms across all time points, and did not drastically change throughout the study. Generally, deterioration in motor function occurs in the more advanced stages of CTS [23]. Although clinical and neurographic signs of median nerve motor damage appear to be poorly correlated to motor symptoms, experimental data suggest that symptoms of pain may modulate motor function [24]. As pain symptoms remained relatively unchanged during the 4 weeks of radioulnar wrist compression intervention, pain modulation on motor function may have mechanistically contributed to patients’ lack of functional improvement in this study.

This study has several limitations. First, the duration of this study was relatively short, due in part to the bulky design of the brace and bulb configuration, which prevented long-term wear. During each bracing session, the subject was required to remain seated with his or her hand resting on a horizontal surface for the duration of pressure application. Future studies can evaluate the effects of longer bracing durations, including overnight wear using a slenderized device that applies pressure and depressurizes automatically. Second, the protocol was not evaluated against a placebo/sham, or standard of care treatment due to the small sample of CTS patients recruited for this study. Future work will explore the extent to which the effects of radioulnar wrist compression intervention surpass those of a placebo and/or standard of care treatment in a larger patient pool. Lastly, although we saw some symptom recurrence during the four-week follow-up period, the extent to which benefits diminish over time is not yet fully understood. It would be worthwhile to investigate the duration of radioulnar wrist compression intervention benefit over an extended follow-up period.

In conclusion, radioulnar wrist compression effectively alleviated sensory symptoms attributable to CTS. In particular, symptom severity scale and tingling and numbness improved by the second week of the intervention. Following radioulnar wrist compression cessation, symptoms of numbness and tingling improved compared to initial baseline measures. Hand motor impairment such as pinch and grasp weakness had a lower frequency across CTS sufferers and did not improve. Particular to patients with mild to moderate CTS, radioulnar wrist compression might be an effective alternative treatment to alleviate sensory symptoms. Future clinical trials are needed to optimize the force application dosage (magnitude, duration, and frequency) for different severity of CTS.

## Data Availability

The datasets used and/or analysed during the current study available from the corresponding author on reasonable request.
